# The Cell Biologist Potential of Cytomegalovirus to Solve Biogenesis and Maintenance of the Membrane Recycling System

**DOI:** 10.3390/biomedicines13020326

**Published:** 2025-01-31

**Authors:** Pero Lučin, Hana Mahmutefendić Lučin

**Affiliations:** 1 Department of Physiology, Immunology and Pathophysiology, Faculty of Medicine, University of Rijeka, Braće Branchetta 20, 51000 Rijeka, Croatia; hana.mahmutefendic@uniri.hr; 2 Department of Nursing, University Center Varaždin, University North, Jurja Križanića 31b, 42000 Varaždin, Croatia

Cytomegalovirus (CMV) is an important pathogen that extensively remodels the nucleus and cytosol of an infected cell to establish a productive infection [1,2,3,4]. Nuclear events include the formation of large structures that are known as nuclear replication centers (NRCs), where viral DNA replication and nuclear capsid assembly occur. Cytosolic events include the complete reorganization of the cytoskeleton and the membrane system (Figure 1). The reorganized membrane system (RMS) of the infected cell involves the relocation of the Golgi into a ring-like configuration that encloses a large perinuclear region containing early endosomes (EEs), recycling endosomes (REs), the trans-Golgi network (TGN), and expanded membrane structures of membrane intermediates at the EE-RE/ERC-TGN interface (Figure 1B) [5,6,7,8]. This structure, which is as large as the nucleus of the infected cell, is referred to as the cytoplasmic assembly complex (cAC) and is likely the site of the final steps of CMV virion assembly, including the envelopment of the tegumented capsids by cellular membranes and the establishment of the pathway for virion egress from the cell [2]. The endoplasmic reticulum (ER) and late endosomes (LEs) are extruded together with the secretory system of the cell from the AC towards the cell periphery (Figure 1B) [2,5,8]. This extensive reorganization of the membrane system, which is often accompanied by a compaction of the cell due to the restructuring of the cytoskeleton, obviously involves a redirection of membrane flux that is difficult to compare with that in the flat cell. Overall, little is known about the transport pathways, membrane flux, and remodeling of membrane organelles in such a reorganized membrane system.

Each cell adapts membrane flux by sequentially cascading membrane domains and incorporating specific domains into organelles to sort membrane proteins and organize membrane-associated physiological processes. Membrane flux is regulated by small Rab-family GTPases and phosphoinositides (PIs) that define the identity of membrane domains on organelles in a cascade or maturation sequence and orchestrate membrane traffic [9]. About 60 different Rab proteins are turned on and off in a cascade of guanine exchange factors (GEFs) and GTPase-activating proteins (GAPs), while the maturation of PIs is controlled by 19 kinases and 28 phosphatases. Activated Rab GTPases associate with membranes and cooperating PI variants, sometimes also with the GTPases of other families (e.g., Arf), and recruit all the necessary components from the cytoplasm to perform the trafficking step and demarcate specific domains on membrane organelles. The recruited effector proteins adjust the lipid composition, balance the dynamics of membrane curvatures, perform membrane tubulation and generate transport intermediates, associate with cytoplasmic complexes and biomolecular condensates, link membranes to cytoskeletal trajectories, define the zip codes for routing of transport intermediates, create a platform for sorting membrane proteins, etc. The duration of the step lasts until shutdown, which leads to the dissociation of the recruited components, but is preceded by the recruitment and activation of the GTPases of the next step. These sequences are organized spatially and temporally according to the needs of each individual cell and its developmental state (i.e., resting and dividing cells), resulting in variation in organelle size and shape. Some of the stages are bottlenecks in the flux, resulting in the constant presence of some organelles and the basic configuration of the membrane system.

During CMV infection, the formation of membrane domains is reorganized to create an efficient environment for virus replication. Reorganization is initiated early in infection, and the basic configuration of the RMS and AC is established in the early (E) phase of infection prior to viral DNA replication and late (L) gene expression [8,10,11,12,13,14]. The functions of the RMS in the E phase are related to the evasion of recognition by the host immune system, reorganization of the cell surface proteome, abrogation of the cellular signaling cascade required for cell cycle progression, maintenance or even amplification of signaling required for viral DNA replication, and amplification of the signaling required for viral gene expression [11,15,16,17,18]. In addition, RMS in the E phase also forms the membrane composition and membrane organelles required for the final cytoplasmic assembly of virions, their packaging into transport organelles, and the establishment of the exit pathway for newly formed virions. Many structural components of virions are later incorporated into the RMS during the L phase of infection after viral DNA replication.

The E virus genes that control the entire process are not known. In human CMV (HCMV)-infected cells, the establishment of the full configuration of the pre-AC takes 48 h or longer [2,14], whereas in murine CMV (MCMV)-infected cells, the basal configuration is rapidly established between 5 and 7 h after the infection of fibroblast-like cells and proceeds until the onset of viral DNA replication, which occurs at 15–16 hpi [8]. An open question in CMV biology is how such extensive reorganization of the cell can be achieved with a limited gene repertoire encoded in the CMV genome. For example, ~5.7% of the MCMV genes are significantly expressed at 2 hpi and a further ~28.4% at 8 hpi [19], at a time when the basic configuration of the RMS is established. A significant proportion of these genes at the edges of the genome are not shared with HCMV and do not contribute to RMS [13]. Since most of these gene products migrate to the nucleus, no more than 3–5% of the MCMV gene products can be used for RMS. In an HCMV study in which 26 genes coding for E and L proteins were investigated using small interfering RNAs (siRNAs), three (UL48, UL94, and UL103) were identified whose silencing influences AC development [7]. These three proteins are the tegument proteins of the virion, which are conserved in all Herpesviridae. In contrast to MCMV, where the proper formation of pre-AC is a prerequisite for DNA synthesis [11], the development of AC in HCMV-infected cells depends on viral DNA synthesis and the expression of one or more L genes, which may explain why the whole process is much slower in HCMV-infected cells [7]. In addition to viral proteins, the biogenesis of AC has been shown to be regulated by microRNAs (miRNAs) encoded by HCMV (miR UL112-1, US5-1, and US5-2) that target the mRNAs of various host proteins involved in the regulation of the cellular membrane system [20].

An extensive spatial reconfiguration of the membrane system may be associated with the reorganization of the cytoskeleton as manifested by cell contraction and cell rounding, which are a prominent cytopathogenic effect of CMV infection. Both HCMV and MCMV target cytoskeletal structures, and microtubule depolymerization disrupts the entire AC structure [7]. However, the formation of some membrane features that characterize the pre-AC of MCMV-infected cells occurs after infection with a recombinant virus that does not express genes responsible for cell rounding [8]. Additionally, pre-AC was frequently observed in cells before complete cell rounding, and AC is developed in HCMV-infected cells without cell rounding.

The hallmark of membrane system reorganization in the establishment of pre-AC and AC is the targeting of the Golgi and the endosomal recycling system at the EE-RE/ERC-TGN interface [8,21]. The identification of small GTPases, PIs, and effector proteins enables the identification of the effector capacities of membrane domains and organelles and is used as an important principle to reconstruct biogenesis and the assembly of membrane organelles within the cell. This type of analysis within the huge cellular area resembling pre-AC revealed the extensive reorganization of membrane domains, expansion of many domains at the EE-RE/ERC-TGN interface, and even the formation of hybrid organelles [5,8,10,11,12,21,22], associated with the alteration of trafficking processes, including the inhibition of endosomal recycling [12,13,20,23]. These processes are regulated by many cellular proteins in a precisely ordered sequence [24], and CMV infection generally alters this sequence, as evidenced by the alteration of their composition at the transcriptional level [25,26,27,28], by proteomic analysis [17,18,29], and redistribution between organelles based on the knowledge of conventional organelle composition [18,30,31].

Structures with an AC-like configuration have been identified in uninfected cells so that pre-AC biogenesis is based on the activation, deactivation, or modification of previously programmed cellular processes related to the organization of the membrane system [6]. One of the processes involved is the extensive tubulation of membranes at the interface of EEs, REs/ERC, and TGN, suggesting that CMV targets the termination of the membrane tubulation process [8,11]. Tubulation coincides with Golgi unlinking and dislocation, and it is unknown whether these processes are coupled. Therefore, it is reasonable to speculate that CMV may disrupt the homeostatic balance between ERC, TGN, and Golgi, leading to the expansion of tubular membranes and extrusion of the Golgi from the cell center to the periphery. Since it is largely unknown what controls the positioning of these organelles around the cell center and maintains their shape, spatial distribution, and size by the incoming and outgoing flux, it can be assumed that CMV again demonstrates its cell biologist potential and points to crucial principles of membrane organelle biogenesis.

The key to identifying CMV targets for membrane expansion within the pre-AC may lie in the rapid unlinking of the Golgi ribbon and its displacement from the cell center that accompanies the expansion of the EE-RE-TGN interface. Indeed, it has been proposed that the Golgi ribbon represents a permanent template that generates transient Golgi stacks and connects them into a functional network with so-called “linker compartments” that represent an intermediate compartment of the secretory pathway and the ERC of the endosomal system comprising events at the EE-RE-TGN interface [32]. Repositioning of the Golgi and centrosomes has been observed in many normal physiological processes, such as cell division and migration and cell differentiation, but also in pathophysiological conditions such as cancer and neurodegeneration [32]. Golgi repositioning has also been linked to microtubule nucleation and autophagy, two processes associated with the AC of infected cells [33,34] and which may be crucial for the assembly of viral exit pathways [35].

Understanding the biogenesis of AC can, therefore, rely, to some extent, on hypothesis-driven research based on the existing knowledge of the composition, maintenance, and physiological alignment of events in the Golgi and linker compartments. However, knowledge is limited and the time frame for these processes is quite long. Therefore, hypothesis-driven approaches, especially those based on the use of genetic tools, should be combined with careful studies of CMV-induced processes using an observation-based approach in advanced techniques of long-term live-cell imaging and the use of fast-acting small molecule inhibitors. It has been shown that it is important to learn from viral infections, not only to understand their biology and pathogenesis, but also to learn about normal cell physiology. This is logical, as viruses have co-evolved with their host over thousands of years, giving them the opportunity to learn which steps are crucial in certain biological processes and giving them enough time to evolve efficient Trojans to adapt essential processes to the needs of their replication. Their discovery highlights the crucial steps in the physiology of the process, and the viruses help cell biologists target the right sequence, which is essential for the next generation of hypothesis-driven research. The cell biologist potential of CMVs has already been highlighted in several reports [33,34,36]. Furthermore, these processes appear to occur much faster in MCMV-infected cells, perhaps due to earlier gene expression, and MCMV could contribute as a cell biologist to the understanding of normal cellular physiology as well as HCMV, an important human pathogen.

CMVs have one of the highest coding potentials of all viruses and thus cause a comprehensive remodeling of the cell. Extensive research over the last decades has identified functions for many CMV coding products. Nevertheless, many of them remained uncharacterized. Technological advances and the development of omics approaches have led to extensive data on CMV-encoded functions and changes in host cell functions, including the complexity of host cell factors that regulate membrane systems at temporal and spatial scales. Single-omics analyses are now evolving into a multi-omics approach [37]. The wealth of data obtained from the analysis of normal cellular processes requires the development of new tools and approaches for multidimensional integration to better understand membrane system physiology, e.g., the in silico modeling of membrane fluxes, membrane domain shaping, biogenesis, and dynamics of membrane organelles, including the heterogeneity of their adaptation to the needs of the cell. Accordingly, incorporating the available information on CMV coding potential and virus–host interactions will not only improve the understanding of CMV biology, but also enhance the cell biologist potential of CMVs in elucidating normal cellular processes. Although this seems to be a daunting task, advances in generative artificial intelligence tools are opening the doors for such approaches. The development of comprehensive maps of the interaction of CMV gene products with the host cell will enable a new generation of hypothesis-driven research that will be an irreplaceable tool for understanding CMV biology.

CMV manipulations of the membrane system of the infected cell are directly related to the pathogenesis of CMV infections [4]. The pathogenesis is also complex and involves productive replication only in some cell types, the establishment of latency, and reactivation from latency to develop either a productive infection or unproductive replication that impairs host functions [38]. The mechanisms for these processes are still incompletely known, and elucidation of the intracellular manipulation of the host cell membrane system is critical to their understanding. Therefore, the multiple and redundant options for the assembly of the membrane system of each cell should be reconciled with the multiple targeting approaches that CMV has developed for the establishment of lytic and latent infections [4,39]. Progress in understanding CMV pathogenesis is related to the understanding of normal physiology and redundancy within intracellular transport pathways, which are also inadequate. Most existing knowledge of CMV is based on the study of fibroblast-like cells, whereas CMV has a much broader cell tropism, and manipulation appears to occur in all the infected cell types [39]. The consequences probably depend on the specificity of the configuration of the membrane system in each individual cell type. Even in relatively homogeneous cell populations, such as the cells in cell cultures, there is a high degree of heterogeneity in the cellular response to infection, as has been observed and documented following infection with other viruses [40,41]. The complexity of heterogeneity is particularly important in CMV infections when it comes to producing viral progeny and finding their way out of the cell. In addition, the manipulation of the membrane system is important for another aspect of pathogenesis, namely the evasion of host control mechanisms such as innate and adaptive immunity. CMV have evolved a significant part of their coding potential to disrupt many facets of the immune system, including antigen presentation, recognition by T and NK cells, and the prevention of inflammatory responses [42].

An important aspect of the interplay between CMV coding potential and the redundancy of host cellular processes, including the reorganization of the membrane system, is the identification of new targets for therapeutic intervention. Conventional antiviral drugs have been developed by targeting essential viral components, but these strategies are insufficient as drug-resistant viruses emerge [1,43]. The successful identification of host cell factors that are essential for CMV pathogenesis but dispensable for the host cell could serve as new targets for antiviral drugs, the so-called host-directed antiviral therapy [43].

Overall, there are many aspects of CMV pathogenesis and host physiology that function at the cellular and higher order level in which CMV may have a cell biologist potential. Therefore, concurrent advances in the study of HCMV and experimental models such as MCMV will be accompanied by advances in understanding the cellular and higher-order physiology as well as the pathophysiology of CMV infection.

## Figures and Tables

**Figure 1:**
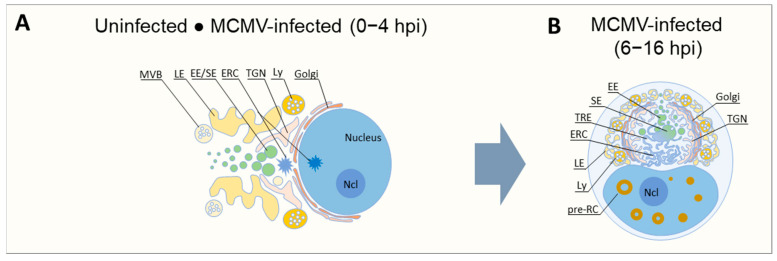
Reorganization of the membrane system in murine CMV-infected cells in the early phase of infection. (**A**) In uninfected fibroblast-like cells in interphase, the Golgi is arranged in a juxtanuclear configuration from the cell center and encircles the nucleus. The linker compartments, the endosomal recycling compartment (ERC), and the intermediate compartment of the biosynthetic pathway (not included) accumulate around the centrosome in the non-compact zone of the Golgi ribbon or under the nucleus. The pericentriolar zone is surrounded by a more central layer containing sorting endosomes (SEs) and the trans-Golgi network (TGN), and a more peripheral perinuclear region containing late endosomes (LEs), early endosomes (EEs), multivesicular bodies (MVBs), and lysosomes (Lys). Large tubular recycling endosomes (REs) are rarely observed as they are highly dynamic, short-lived structures. (**B**) In MCMV-infected cells, the configuration of the membrane system of uninfected cells is mainly maintained during 4–5 h post-infection (hpi). This period is characterized by the formation of 6–8 pre-replication centers (pre-RCs) in the nucleus. At 5–7 hpi, extensive reorganization of the membrane system begins, in which the Golgi detach from the juxtanuclear region, expand, and shift into a ring-shaped structure, combined with the expansion of EEs, REs, TGN, and ERC in the pericentriolar region, resulting in a basic structural configuration known as pre-AC. Later in the early phase of infection, up to 16 hpi, when the replication of viral DNA begins, the central region surrounded by the Golgi stacks (known as the inner pre-AC) continues to mature and expand. The hallmark of the inner pre-AC is the expansion of tubular membrane domains (shown in blue), including tubular recycling endosomes (TREs), domains of the ERC, and domains at the EE-TGN interface. Late endosomes (LEs), multivesicular bodies (MVBs), lysosomes (Lys), and the endoplasmic reticulum (not included) are mainly extruded from the pre-AC region towards the cell periphery. Ncl, nucleolus.
